# Clinical characteristics and suicidal ideation as predictors of suicide: prospective study of 1000 referrals to general adult psychiatry

**DOI:** 10.1192/bjb.2024.67

**Published:** 2025-12

**Authors:** David Hayward, Blair Johnston, Donald J. MacIntyre, Douglas Steele

**Affiliations:** 1 NHS Lothian, Livingston, UK; 2 NHS Greater Glasgow and Clyde, Glasgow, UK; 3 University of Edinburgh, Edinburgh, UK; 4 NHS Research Scotland, Glasgow, UK; 5 University of Dundee, Dundee, UK; 6 NHS Tayside, Dundee, UK; 7 University of St Andrews, Fife, UK

**Keywords:** Suicide, suicidal ideation, general adult psychiatry, mortality, risk assessment

## Abstract

**Aims and method:**

Questions often follow the suicide of someone who presented to general adult psychiatry (GAP) when expressing suicidal thoughts: ‘Why were they not admitted, or managed differently, when they said they were suicidal?’ Answering these questions requires knowledge of the prevalence of suicidal ideation in patients presenting to GAP. Therefore, we determined the general clinical characteristics, including suicidal ideation, of a large sample of patients presenting to a GAP emergency assessment service or referred as non-emergencies to a GAP service.

**Results:**

Suicidal ideation was very common, being present in 76.4% of emergency presentations and 33.4% of non-emergency referrals. It was very weakly associated with suicide, varied between different diagnostic categories, and previous assessment by GAP did not appear to affect it. The suicide rate during the contingent episode of care was estimated as 66 per 100 000 episodes.

**Clinical implications:**

This, and other evidence, shows that suicide cannot be predicted with an accuracy that is useful for clinical decision-making. This is not widely appreciated but has serious consequences for patients and healthcare resources.

Suicidal ideation is often assumed to be on a continuum that can culminate in suicide and, in some cases, be indicative of mental disorder. In the UK National Health Service (NHS), services, especially GAP, are expected to prevent suicide by attending to people who report suicidal ideation to (a) assess whether they have a mental disorder, (b) try to predict whether they are at particularly high risk of suicide (the ‘risk assessment’) and (c) make provisions to try to prevent suicide. Therefore, when a patient who had been in contact with NHS mental health services expressing suicidal ideation dies by suicide, it is often asked ‘why was the suicidality not recognised?’ and ‘why was the suicide not prevented?’. This is presumably, at least in part, because suicidal ideation is thought to be unusual and, therefore, pertinent. We provide evidence-based answers to these questions, including by reporting for the first time the prevalence of suicidal ideation in both emergency presentations and non-emergency referrals to a GAP service. The implications for clinical decision-making are discussed.

## Method

Ethical approval for the study was obtained (London Riverside Research Ethics Committee (REC) reference 22/LO/0473).

### Study sample

Between March 2022 and February 2023, the NHS hospital electronic record system recorded 3045 patient presentations to a GAP service with 6 full-time equivalent consultants, based in a district general hospital in Scotland, serving an urban population of about 57 000 and a surrounding more rural catchment area population of about 125 000. The patients had either presented to the emergency assessment service, which sees patients referred by accident and emergency (A&E) departments, police and general practitioners (GPs) for same day assessment, or had been referred routinely to the weekly written referral meetings, which considers non-emergency referrals from GPs and NHS therapists at the local primary care mental health service. During that period, staff involved in the study collected data prospectively until 1000 referrals had been reached. Crucially, to ensure this was a representative unfiltered sample of all GAP referrals, there were no exclusion criteria and all referrals were included during the data collection periods. The overall data collection period was briefly interrupted on a few occasions by temporary staffing limitations.

### Data collection

For the emergency assessment service patients, staff conducted their assessments as usual and recorded this on the hospital electronic system. Then they recorded data pertinent to this study on a separate pro forma sheet, without noting patient identifiable details. The presence of suicidal ideation was recorded if the patient volunteered, or affirmed when asked, that they had been thinking about suicide or wanting to be dead. For the written referral meeting patients, the attendant consultant psychiatrist recorded data for the study as the referrals were discussed. Suicidal ideation was recorded if the referral letter stated that the patient had been thinking about suicide or wanting to be dead. The study was entirely observational so did not influence the way patients were appraised.

The prevalence of suicidal ideation, the associated provisional clinical categories and whether the patient had been assessed by GAP before were noted by the consultant psychiatrist for written referrals and by the nurse practitioners and psychiatrists who conducted the emergency assessments.

In August 2023, 6 months after data collection ended, when all the emergency assessment service episodes of care that were informed by the surveyed assessments would have concluded, we recorded how many patients were formally recorded by the Procurator Fiscal Service to have died by suicide from the 3045 total presentations.

### Provisional clinical diagnoses

A full psychiatric diagnostic assessment usually includes information from any past presentations to mental health services, information gleaned during indirect assessments and information from informants who know the patient well. Such comprehensive information takes time to collect and is usually impractical in an acute assessment situation. Hence, in NHS practice, initial provisional diagnoses tend to be broad, so are here called initial ‘clinical categories’. This is reflected by the provisional clinical categories used in the present study, which were, for each patient, one or a combination of (a) ‘low mood’ (ICD-11 Block L1-6A6 excluding mania/hypomania), (b) ‘mania’ (ICD-11 Block L1 only mania/hypomania), (c) ‘alcohol-related’ (ICD-11 6C40), (d) ‘drug-related’ (ICD-11 Block L2-C64 excluding alcohol), (e) ‘personality issues’ (ICD-11 Block L1-6D1), (f) ‘post traumatic’ (ICD-11 Block L1-6B4), (g) ‘psychosis’ (ICD-11 Block L1-6A2), (h) ‘problem eating’ (ICD-11 Block L1-6B8), (i) ‘cognitive’ (ICD-11 Block L1-6D7 and Block L2-6D8) and (j) ‘neurodevelopmental’ (ICD-11 Block L1-6A0).

The definition of suicidal ideation used here (thinking about suicide or wanting to be dead) is broad, encompassing everything from being resigned to not waking up to being committed to self-annihilation. This is deliberate because nowhere is suicidal ideation defined, but the term is often used and considered salient.

### Statistical analysis

Exploratory statistical analyses, and tests of the null hypothesis of no association between variables, were done using the chi-squared text on JASP (version 0.17.3 for Windows).^1^

## Results

During the survey period, the emergency assessment service assessed 847 patients and collected data on 326. Within the subsequent episode of care administered by the emergency assessment service, one patient died by suicide. During the same period, the non-emergency referral meetings considered 2198 referrals and collected data on 674. Within the subsequent episode of care, one patient died by suicide. Thus, two patients died by suicide out of the 3045 assessed by the whole GAP service during the study period: a suicide rate of 66 per 100 000 (2 suicides/3045 assessments × 100 = 0.065%, or 66 per 100 000).

Suicidal ideation was extremely common in most of the provisional clinical categories, with alcohol-related presentations having the strongest association (χ^2^ = 40.951, *P* < 0.001) and problem eating presentations having the weakest association (χ^2^ = 3.968, *P* = 0.046). There was no significant association between suicidal ideation and mania (χ^2^ = 1.198, *P* = 0.274) or the post-traumatic category (χ^2^ = 0.022, *P* = 0.883). In total, 34% of emergency assessments and 37% of non-emergency referrals could be allocated two or more provisional clinical categories. Adjusting for these multiple diagnoses, suicidal ideation (*n* = 474) was recorded as present for 76.4% of emergency presentations (*n* = 326) and 33.4% of non-emergency referrals (*n* = 674).

Suicidal ideation was more strongly associated with assessments done by the emergency assessment service, and less so with referrals considered at the non-emergency referral meetings (χ^2^ = 164.359, *P* < 0.001). However, the positive predictive value (PPV) of suicidal ideation for suicide in both groups was extremely low and the odds ratios (ORs) were not statistically significant, despite the very large (1000) sample size. For the emergency assessment service, the PPV was 0.16% and the OR was 0.92 (95% CI 0.04–22.81, *P* = 0.96); for written referrals, the PPV was 0.15% and the OR was 6.01 (95% CI 0.24–148.04, *P* = 0.27); and for all GAP, the PPV was 0.16% and the OR was 6.07 (95% CI 0.29–126.44, *P* = 0.25).

Therefore, although the prevalence of suicidal ideation differs substantially between patients referred as emergencies and those referred routinely, the absolute risk of suicide in both groups was extremely low (emergency assessment service: 0.12%; written referral meetings: 0.05%; whole GAP service: 0.07%). Also, suicidal ideation was not significantly different in patients who had been previously assessed compared with patients who were assessed for the first time (χ^2^ = 4.836, d.f. =2, *P* = 0.089). Patients referred as emergencies to GAP were more likely to have been assessed previously by GAP (59.8% *v.* 37.5%).

Details of the assessment data are summarised in Tables 1–3.

**Table 1:** Clinical categories and suicidal ideation

Provisional clinical category^a^	Patients presenting to emergency assessment service (*n* = 326), *n* (%)	Patients with written referrals (*n* = 674), *n* (%)	Total patients in GAP service (*n* = 1000), *n* (%)	Patients with suicidal ideation (*n* = 474), *n* (%)	Strength of association of clinical category with suicidal ideation, χ^2^
Low mood	63 (19.33%)	133 (19.73%)	196 (19.60%)	128 (65.3%)	31.189, *P* < 0.001
Mania	10 (3.07%)	24 (3.56%)	34 (3.40%)	13 (38.2%)	1.198, *P* = 0.274
Anxiety	52 (15.95%)	248 (36.8%)	299 (30.0%)	124 (41.5%)	6.111, *P* < 0.013
Alcohol-related	75 (23.01%)	14 (2.08%)	89 (8.9%)	71 (79.8%)	40.951, *P* < 0.001
Personality	92 (28.22%)	150 (22.26%)	241 (24.2%)	143 (59.3%)	18.004, *P* < 0.001
Trauma	47 (14.42%)	170 (25.22%)	217 (21.7%)	102 (47%)	0.022, *P* = 0.883
Psychosis	23 (7.06%)	39 (5.79%)	62 (6.2%)	12 (19.4%)	20.922, *P* < 0.001
Drug-related	76 (23.31%)	16 (2.37%)	92 (9.2%)	60 (65.2%)	12.833, *P* < 0.001
Problem eating	0	17 (2.52%)	17 (1.7%)	4 (23.5%)	3.968, *P* = 0.046
Cognitive	1 (0.31%)	28 (4.15%)	29 (2.9%)	3 (10.3%)	16.489, *P* = 0.001
Neurodevelopmental	8 (2.45%)	89 (13.21%)	97 (9.7%)	26 (26.8%)	18.360, *P* = 0.001

GAP, general adult psychiatry.

a. Some patients were assigned to more than one clinical category.

**Table 2:** Had the patient been assessed by general adult psychiatry (GAP) before?^a^

Previous assessment	Patients presenting to emergency assessment service, *n* (%)	Patients with written referrals, *n* (%)	Total patients in GAP service, *n* (%)
Yes	195 (59.8%)	253 (37.5%)	448 (55.1%)
No	130 (39.9%)	421 (62.5%)	551 (44.8%)
Not recorded	1	0	1
Total	326 (100%)	674 (100%)	1000 (100%)

a. The strength of association between previous GAP assessment and suicidal ideation χ^2^ = 4.836, d.f. = 2, *P* = 0.089.

**Table 3:** Multiple provisional clinical categories (comorbidities)

Initial diagnoses, *n*	Patients presenting to emergency assessment service, *n* (%)	Patients with written referrals, *n* (%)	Total patients in GAP service, *n* (%)
0 (patient did not meet criteria for any provisional clinical category)	25 (7.67%)	23 (3.41%)	48 (4.8%)
1	190 (58.28%)	401 (59.5%)	591 (59.1%)
2	78 (23.93%)	223 (33.09%)	301 (30.1%)
3 or more	33 (10.12%)	27 (4.01%)	60 (6%)
Total	326	674	1000

## Discussion

### Association between suicidal ideation and suicide

Most NHS psychiatrists work in GAP, and suicide risk assessment forms a substantial part of the GAP workload.^2^ However, the prevalence of suicidal ideation in people who present to GAP had not been previously reported.

In the present study, the suicide rate during a new episode of care was 66 per 100 000 episodes. In comparison, between 2010 and 2020, the rate of suicide among mental health service patients across the UK was 48 per 100 000,^3^ whereas in 2021, the annual suicide mortality rate in the general Scottish population was 13.7 per 100 000 people.^4^ Therefore, our data corroborate reports of a higher rate of suicide in people known to mental health services.

Here, 47.4% of patients referred to GAP reported suicidal ideation when they were assessed (including 76.4% of patients referred as emergencies), yet suicide during the contingent episode of care was very rare. Our study then is in general agreement with others on risk assessment. For example, a case–control study of risk factors for suicide among in-patients and recently discharged out-patients with affective disorders reported 48% of patients having ‘suicidal thoughts at admission’,^5^ and a meta-analysis of the predictive utility of suicidal ideation for suicide among GAP patients during the comparatively high-risk-for-suicide in-patient and immediate post-discharge phases also determined an extremely low PPV, even with a long average follow-up period of 9.1 years (pooled PPV among 29 cohort studies was 1.7%, 95% CI 0.9–3.2).^6^

The negative predictive value (NPV) of suicidal ideation for suicide is also extremely low, which is to say that the absence of suicidal ideation is not an assurance of safety. For example, one retrospective study found that almost 80% of psychiatric in-patients who died by suicide during that admission had denied suicidal thoughts in their last verbal communication.^7^

Further international evidence of an extremely weak association between suicidal ideation and suicide is provided by results from the US National Survey on Drug Use and Health, when compared with the Centers for Disease Control and Prevention mortality records. In that 2021 survey, only 1.6% of participants aged 65 and over affirmed ‘thinking seriously about killing themselves in the previous 12 months’, despite that group including the highest annual rate of suicide (40 per 100 000 for White males aged 75 and over). This contrasts with the 18- to 25-year age group, who had a far higher prevalence of suicidal ideation at 6%, but a much lower rate of suicide (17.5 per 100 000 for males and 4 per 100 000 for females).^8^

### Association between other presumed risk indicators and suicide

The objective evidence then is that suicidal ideation is extremely common in people presenting to GAP, and it is very weakly associated with suicide at the group level. This is also true for other presumed risk indicators for suicide. For example, meta-analysis of the predictive utility of various suicide risk assessment scales (Beck Hopelessness Scale, Suicide Intent Scale, and Scale for Suicide Ideation) and clinical risk factors (including alcohol misuse, concomitant physical health ailments, unemployment and previous self-harm) after self-harm showed them not to be clinically useful.^9,10^ Furthermore, meta-analysis has also concluded that ‘suicidal behaviours’, including ‘suicide attempts’, which might be considered strong indicators of suicide risk, are no more strongly associated with suicide than suicidal ideation.^11^ Despite this, association statistics continue to be accumulated and, although they can appear compelling (for example, one study found the age-adjusted risk of suicide in the first year following self-harm to be 49 (95% CI 43–57) times greater than in the annual general population^12^), they do not help the practitioner determine which of the people they assess will die by suicide, when or what might keep them safe.

Our data, and these meta-analyses, illustrate the fundamental problems with attempting to predict extremely low-probability events with undiscerning assessments. Commensurately, it has been concluded that ‘the idea of risk assessment as risk prediction is a fallacy […] We are simply unable to say with any certainty who will and will not go on to have poor outcomes’.^9^ Our study extends this conclusion to patients at the point of assessment by GAP. At that juncture too, suicidal ideation does not predict suicide with a specificity, nor within a time frame, that is clinically useful.

### Suicidal ideation does not necessarily imply mental disorder

Additionally, it should not be assumed that suicidal ideation implies the presence of a mental disorder that requires the involvement of GAP services. To illustrate, the prevalence of suicidal ideation varies widely between regions (for example, from 2.09% in Beirut, Lebanon, to 18% in Christchurch, New Zealand), but the rates of psychiatric disorders do not,^13^ and, further, the prevalence of suicidal ideation does not mirror that of depressive disorders.^14^

Unless suicidal ideation is a consequence of a psychiatric disorder, GAP services do not have a unique contribution to make to reducing any associated risk of suicide. Qualitative evidence for this includes that when GAP staff were asked to consider in retrospect whether there was anything distinctive about their patients who died by suicide, what was often reported was a significant clinical improvement, but the persistence of concomitant stress factors.^15^ Social workers and third-sector organisations are better placed than GAP services to address social factors, so they may be more likely to reduce the subjective burden of suicidal ideation and the risk of suicide, including in emergencies. Indeed, ‘informal social support’ was considered a ‘particularly promising broad-based intervention’ after a Scottish Government commissioned systematic review of interventions to prevent suicide and suicidal behaviour.^16^

Despite this, the National Institute for Health and Care Excellence (NICE) still recommends that a ‘mental health professional’ performs a ‘risk formulation’ after an occasion of self-harm (but not a ‘risk assessment’, which is, presumably, different because it is constrained by a tool or scale). What a risk formulation should entail is only specified in vague social terms, such as ‘historical experiences, recent problems, and existing strengths and resources’.^17^ Social variables may indeed correlate statistically with suicide in large enough samples, but significant group- and population-level associations do not translate into useful predictors at an individual patient level, and they say nothing about how useful a particular therapeutic response might be.

Mistakenly inferring or assuming that studies reporting association statistics have predictive utility contributes to an unrealistic understanding of what suicide risk assessments can achieve^18^ and this has serious consequences for patients and healthcare resources. Precise terminology is required: when only association statistics have been undertaken, it is important to avoid using terms with no clear definition or meaning, such as ‘predictive value’ and ‘predictive power’ or ‘risk assessment’ and ‘risk formulation’.^19–21^

Uniquely, GAP services have the potential to contain people in hospital, but it would be numerically impossible to admit for further scrutiny all those who are assessed with suicidal ideation (approximately 50% of people referred to GAP throughout the UK would have to be admitted). Further, there is little evidence that GAP ward admissions can prevent suicide in general. For example, a recent study of psychiatry in-patients concluded that the majority of those who died by suicide were considered very unlikely to do so, including 14% having no ‘risk factors’ and 30% having only one ‘risk factor’. False-positive rates like this render such assessments clinically useless.^22^

Finally, it could also be that hospital-based care, especially compulsory treatment, is in itself dangerous for people who are thinking about suicide because it could compound those feelings of defeat, humiliation and entrapment that are considered motivators for suicide.^23^ Evidence for ‘nosocomial suicide’ is that patients who received psychiatric medication had 5.8 times the risk of suicide; patients who received out-patient psychiatric treatment had 8.2 times the risk of suicide; patients who had contact with emergency psychiatric services had 27.9 times the risk of suicide; and patients who were admitted had 44.3 times the risk of suicide; and the magnitude of these risk ratios greatly exceeds both the risk of suicide associated with major psychiatric disorder and the presumed clinical risk factors for suicide among admitted patients. This could suggest that current medical practice in the context of suicidal ideation increases the risk of subsequent suicide.^24^

However, not all patients are the same and medical interventions invariably incur both potential benefits and harms: there has long been a debate in GAP about the benefit/harm ratio of NHS interventions, including compulsory detentions, for people with personality disorders unresponsive to medications and acute psychotherapies and who have repeatedly hurt themselves, usually in response to interpersonal difficulties. In contrast, for patients with severe and enduring psychiatric illnesses, such as schizophrenia or psychotic depression, driven to kill themselves by psychotic symptoms but who respond to medications, the benefit/harm ratio of GAP interventions, including compulsory detentions, is far more likely to be understood by society, the patient's family and, most crucially, the patients themselves when recovered.

### Limitations

The broad provisional clinical categories used in this study reflect the limited information available at the time of referral and preliminary assessment, but this is usual in NHS medical practice. The 6-month duration of the study is a limitation and annual mortality rates (the usual measure) cannot be reported. However, the predictive utility of suicidal ideation for subsequent suicide during the contingent episode of care can be reported and this is a more clinically useful statistic (in this context, a meta-analysis of similar patient populations concluded that the duration of follow-up does not significantly moderate the predictive utility of suicidal ideation for suicide^6^). Suicidal ideation was significantly associated with assessments performed by the emergency assessment service, but less so with referrals considered at the weekly non-emergency written referral meeting. This could be due to the differences in how suicidal ideation was determined by each service. Optimally, this study would have been done as a large multi-site study to capture any regional variations, and we suggest this with the national audit recommendation that follows.

### Clinical implications and recommendation

Every few years there is debate in the media about the suicide of a person who had been in contact with GAP and was expressing suicidal thoughts, and a recent NHS Resolution review highlighted ‘risk assessment’ as a prominent theme in suicide-related legal claims.^25^ The inevitable question is ‘why did services not admit the person to hospital when they said they were thinking about suicide?’

Our study goes some way to answering this: (a) it is impossible, given the prevalence of suicidal ideation in people presenting to GAP, and potentially harmful to admit every patient who presents with suicidal ideation; (b) suicide cannot be predicted with a specificity and sensitivity, nor within a time frame, that is clinically useful (the group-level associations between suicide and psychosocial variables that have been reported provide only probability estimates which translate poorly, if at all, to the individual, especially in relation to such rare events).

The fact that suicide risk prediction is ineffective has been asserted before, further to the highest standard of evidence.^26^ Additional examples to those already discussed include a meta-analysis of more than 3000 risk factors for suicidal thoughts and behaviours which concluded that ‘prediction was only slightly better than chance’,^27^ including, for example, prior psychiatric hospital admissions and suicide attempts. Moreover, an examination of 64 models of suicide prediction found that the ‘accuracy of predicting a future [suicide] event is near 0’.^28^ What needs to be examined is not the utility of risk predictors, but the efficacy of risk management plans to reduce distress and provide relief.

In practice the NHS does not to admit to hospital the vast majority of the large number of people presenting to GAP with suicidal ideation. The main reason is that admission is unlikely to be beneficial for most people with suicidal ideation. However, every few years every clinician working in a GAP service with a usual clinical workload will likely have a patient die by suicide because they interact with such a large number of people with suicidal thoughts. This may not be well-known outside of GAP, and the mismatch could contribute to the trauma GAP staff experience in the aftermath of a suicide.^29^

The National Confidential Inquiry into Suicide and Safety in Mental Health (NCISH)^3^ does not record information on suicidal ideation for people presenting to GAP, despite NHS staff spending a great deal of time attempting to assess its significance for individual patients. An audit of the management of suicidal ideation in GAP could provide a baseline of current custom and evidence to support quality improvement and transparent evidence-based practice.

## Data Availability

The data that support the findings of this study are available from the corresponding author on reasonable request.

## About the authors

**David Hayward** is a consultant psychiatrist (adult) with NHS Lothian at St John's Hospital, Livingston, UK. **Blair Johnston** is a clinical scientist, MRI Physics, in the Department of Clinical Physics and Bioengineering, NHS Greater Glasgow and Clyde, Glasgow, UK. **Donald J. MacIntyre** is an honorary reader in the Centre for Clinical Brain Sciences, University of Edinburgh, Edinburgh, UK, a consultant psychiatrist with NHS Lothian at the Royal Edinburgh Hospital, Edinburgh, UK, and Director of the NHS Research Scotland Mental Health Network, Edinburgh, UK. **Douglas Steele** is a clinical professor and Professor of Neuroimaging in the Division of Neuroscience, University of Dundee, Dundee, UK, an honorary consultant psychiatrist with NHS Tayside, Dundee, UK, and an honorary professor at the University of St Andrews, Fife, UK.
